# Supplementary material for: Are carbon emissions trading and green financial instruments synergistic? -Comprehensive quantitative research based on content analysis
Source: PLoS One. 2024 Mar 7;19(3):e0298601. doi: 10.1371/journal.pone.0298601 (PMC10919601; doi:10.1371/journal.pone.0298601)
Supplement: S1 Data — (ZIP) [file pone.0298601.s001.zip › Data/地级市Z_F工作报告.zip!/║■─╧/╒┼╝╥╜τ╩╨/└┤╫╘╣1⁄2╓┌║┼í╛┬φ┐╦╩2╛▌═°í┐_2012.docx]

各位代表：

　　现在，我代表市人民政府向大会作工作报告，请予审议，并请各位政协委员及其他列席人士提出意见。

　　一、2011年工作及&ldquo;五年大变样&rdquo;建设回顾

　　2011年既是&ldquo;十二五&rdquo;发展的开局之年，又是&ldquo;五年大变样&rdquo;建设的收官之年。面对国内外发展环境复杂多变和要素配置趋紧等严峻挑战，全市上下在市委的统一领导下，深入贯彻落实科学发展观，按照中央和省委、省政府的决策部署，紧扣建设世界旅游精品和富民强市总目标，踩准节奏，找准路径，奋力拼搏，较好地完成了全年经济社会发展各项目标任务。

　　一是经济发展速度效益同步提升。2011年，预计（下同）全市实现生产总值295亿元，比上年增长14%；完成固定资产投资145亿元、社会消费品零售总额98亿元，分别增长20%和18%；实际利用外资、内资分别增长17.1%和10%；财政总收入（含基金收入）达到31亿元，其中一般预算收入达到17.3亿元，分别增长27.9%和21.1%；城镇居民人均可支配收入达到14039元、农民人均纯收入达到4108元，分别增长10.5%和12%；居民消费价格涨幅控制在5%左右；节能减排完成省定目标任务。

　　二是旅游发展实现新突破。旅游市场竞争力日益增强，景点接待游客总量达到3000万人次，旅游总收入达到160亿元，分别增长25%和28%。过夜游客达到1330万人次，增长33%。东南亚客源市场迅速成长，境外游客接待量达到175万人次，增长18%。天门山晋升为国家5A级景区，黄龙洞注册&ldquo;中国驰名商标&rdquo;，翼装飞行穿越天门洞被评为近两年中国最具影响力的十大营销事件。武陵源区位居全省旅游强县之首。

　　三是产业结构调整步伐加快。一批高档酒店建成营业或开工兴建，溪布街、市博物馆基本建成，体育生态公园、贺龙体育中心等项目建设加快。全市完成工业投资39亿元，规模工业、园区工业增加值分别增长20.5%和70.3%，工业经济效益综合指数上升47个百分点。服务业增加值增长16.5%，华天商贸城、逸臣盛世商业中心等大型商务项目落地兴建。农业结构调整加快推进，蔬菜、油茶、烟叶、茶叶、畜禽、大鲵等生产规模和产业化水平提高。粮食高产创建成效明显，总产达到59.5万吨。

　　四是城乡基础设施建设扎实推进。城市控制性详细规划、专业专项规划和土地利用总体规划编制全面完成，市城区贸易路等7条城市主次干道和鸬鹚湾大桥建成通车，古人堤、庸都园、回龙观公园等绿化景观建设、两区&ldquo;穿衣戴帽&rdquo;和36条小街小巷整治改造完成，溪北路等12条主次干道启动建设。张花高速公路路基工程基本完成，张沅公路南段、S304慈利段建成通车，市中心汽车站投入运营，锦苏特高压输电线路、城乡电网、通信网络建设与改造计划全面实现。农村交通、水利、清洁能源等基础设施投入力度加大，农民生产生活条件进一步改善。

　　五是民生改善取得显著成效。42项省市为民办实事工程全面完成，新增城镇就业1.05万人、农村劳动力转移就业2.32万人、各类社会保险参保70.21万人，开工建设和改造保障性安居住房1.2万多套（户），退休职工养老金、职工最低工资、城乡低保补助和农村五保供养标准全面提高，桑植县参合农民乡镇住院实行全报销。&ldquo;科技市厅会商&rdquo;项目启动实施，义务教育合格学校、校舍安全工程、农村公办幼儿园等教育基础设施建设全面推进，城乡医疗卫生条件不断改善，人民群众文化体育活动日趋活跃，人口和计划生育工作实现省定目标。生态市创建深入推进，城乡生态环境质量进一步提高。

　　六是发展环境不断优化。旅游综合改革试点正式启动，争取省委、省政府出台了27条支持政策。荷花机场升级为国际空港。各级政府加快转变职能，政务服务效能不断提高。国有资本经营预算等制度规范相继建立，土地资源储备和配置工作有力，供销等体制改革积极推进，小额贷款公司挂牌运行，质量兴市和标准化战略启动实施，社会信用体系建设步伐加快。安全生产、食品药品农产品安全和市场价格监管力度加大，社会应急管理和信访维稳工作扎实有效，公众安全感指数排名全省第三。

　　一年来，全市上下凝心聚力，埋头实干，监察、审计、统计、编制、工商、税务、商业保险、粮食、气象、外事侨务、贸促、民族、宗教、残联、移民、对台、档案、史志、无线电管理、新闻媒体等各方面工作也都取得了可喜的成绩。

　　各位代表，随着2011年各项工作的收官，我们今天可以豪迈地说：&ldquo;三年有改观五年大变样&rdquo;建设目标已经基本实现！令人振奋的成就，是在我们相继遭遇特大冰雪冻雨灾害、汶川大地震、甲型H1N1流感、国际金融危机等多重严峻考验情况下取得的，殊为不易，充满着艰辛，充满着奋斗！是全市各族人民在党中央、国务院和省委、省政府的正确领导下，在市委的统一领导下，共克时艰，砥砺前行，化危为机，奋力拼搏的辉煌业绩！这五年，是我市建市以来综合经济实力增长最快、固定资产投资最多、城乡面貌变化最大、民生改善最明显的时期之一，也是广大人民群众反映最好的时期之一。五年间，全市旅游品牌形象大变样，城乡面貌大变样，经济发展格局大变样，民生环境大变样，人民群众思想观念大变样，实现了前所未有的大发展。

　　&mdash;&mdash;经济实力显著增强。全市生产总值年均增长14%，五年实现翻番，三次产业结构由16.4︰24.2︰59.4调整为13.6︰24.7︰61.7。与2006年相比，城乡居民收入水平分别提高66.7%和72%，旅游总收入、工业增加值、服务业增加值、社会消费品零售总额、实际利用内资翻了一番多，财政总收入、固定资产投资、实际利用外资增长两倍多，进出口总额翻了两番多，农村贫困人口累计减少11.2万人，两区两县人均GDP全面突破一万元。

　　&mdash;&mdash;城市面貌焕然一新。全市城镇化率由34%提高到42%，市城区建成区面积由20.5平方公里扩大到28.2平方公里，常住人口从20.5万人增加到24万人。城市道路骨架基本形成，服务功能逐步配套，格调和品位明显提高，集聚力和辐射带动力不断增强。国家旅游综合改革试点城市、联合国世界旅游可持续发展观测点、世界特色魅力城市200强、中国最具海外影响力城市、全国社会治安综合治理优秀市、全国&ldquo;五五&rdquo;普法先进地市、全国&ldquo;双拥&rdquo;模范城、湖南&ldquo;文化强省&rdquo;先进市等桂冠，成为全市新的耀眼名片。

　　--旅游转型明显加快。单一的观光旅游格局已经突破，新型旅游业态开始呈现。铂尔曼、青和锦江等一批高档酒店相继建成，五星级酒店实现零的突破。文化旅游跃居全省塔尖，国际乡村音乐周被誉为"巧用软实力提升国家形象的一次成功实践"，《天门狐仙&middot;新刘海砍樵》等大型演艺节目已成为游客目的性消费项目。"张家界地貌"、"张家界空气"概念获得广泛认同，"平安满意张家界"形象不断深入人心。旅游品牌影响力和美誉度显著提升，旅游市场竞争力全面增强，境外客源国和地区从25个发展到52个。对外交流合作走在全省前列，国际友好城市合作关系由1个发展到6个，并获得了外国人来华访问邀请权。

--工业经济快速成长。全市工业、规模工业增加值比2006年分别增长1.2倍和1.7倍，五年年均分别增长17.3%和21%。全部工业增加值占GDP的比重由17.8%上升到20.9%。规模工业企业由94家发展到143家，年产值过亿元企业由2家发展到41家。园区建设成效显著，入园企业发展到78家，园区工业增加值占全部工业增加值的比重由2.5%上升到24%。市开发区建成面积达到4平方公里，已经展现出工业新城风貌。

--农业结构有效调整。城市农业、旅游农业、品牌农业和生态农业联点成片发展，城郊60%的耕地已退出传统种植，标准化规模养殖比重超过35%，市城区蔬菜、猪肉自给率分别达到70%和45%。市级以上农业产业化龙头企业由34家发展到60家，联接生产基地由35万亩发展到70万亩。农民专业合作组织由41个发展到311个；乡村旅游和休闲农业企业由160家发展到600家。农业标准化生产认证面积达到200万亩，无公害、绿色、有机农产品和国家地理标志保护产品认证达到94个。全市农业增加值达到40亿元，五年年均增长4.8%。

--现代服务业加快发展。五年间，全市服务业增加值年均增长15.7%，比预期目标高0.7个百分点，占GDP的比重由59.4%上升到61.7%。社会消费品零售总额年均增长18.1%，比预期目标高3.1个百分点。金融机构存、贷款余额分别突破300亿元和200亿元，五年分别增长1.9倍和1.5倍；房地产开发年度投资超过30亿元，五年净增5.8倍；现代物流业产值年均增长20%以上，物流企业发展到90余家，梅尼成长为全省第三大零售连锁企业。

--基础设施条件大为改观。五年累计完成固定资产投资567亿元，是建市后前18年总和的1.6倍。相继完成荷花机场扩建一期工程、石怀铁路电气化改造，建成新火车站、中心汽车站和张桑、阳龙、张联、慈石、朱贺公路，新增等级公路4128公里，实现市治到区县一小时通达、村村通公路、乡乡通油路目标。建成长潭河电站等一批能源和水利项目，新增发电装机53万千瓦，解决农村34.13万人安全饮水困难，农村电网改造覆盖面达到71%，耕地有效灌溉率达到53%，市、县城区防洪保安能力分别达到50年和20年一遇。建成电子政务外网平台，市、县城区实现3G网络和有线电视数字化覆盖，农村实现村村通电话、乡乡通宽带。

--社会建设和民生保障全面加强。五年来，全市累计投入为民办实事资金42亿元，共办成142项省定实事和22项市定实事；累计投入城乡就业和社保资金33.2亿元，新增城镇就业岗位3.9万个，实现基本养老和医疗保险城乡全覆盖、城乡低保应保尽保、乡乡镇镇有敬老院目标，解决2.76万户中低收入家庭的住房困难。义务教育阶段学生实现免费就学，"普九"债务基本化解，中小学D级危房基本消除。医药卫生体制改革稳步推进，基本药物制度实现基层医疗卫生机构全覆盖，农民看病负担明显减轻。乡镇综合文化站全面建成，广播电视综合覆盖率达到96.5%。可以这么说，民生需求从生存型已逐步步入发展型阶段。全面实现节能减排预期控制目标，全市森林覆盖率、城市空气质量优良率比2006年分别提高2.65和7.7个百分点。武陵源区业已建成国家级生态示范区。

各位代表，"三年有改观五年大变样"建设是一步一个脚印走过来的，既定目标任务绝大多数已经胜利实现，很多指标超额完成，但是，由于国际国内经济环境复杂多变等客观因素影响，旅游总收入、旅游的外向程度、城市绿化率等少数指标还没能达到预期；规划建设的重点项目中，因国家和省里投资放缓，张花高速公路等一些项目尚未竣工，或未能开工建设，仍需继续努力。

各位代表，五年的历程极不寻常，五年的成就激励人心。这是党中央、国务院和省委、省政府正确领导，历届市委、市政府给我们奠定良好基础，各方面热情关怀、大力支持的结果。在此，我代表市人民政府，向对张家界发展做出卓越贡献的历届市委、市人大、市政府、市政协的老领导、老同志，向全市各族人民，向驻张部队、武警官兵和政法干警，向各民主党派、工商联、人民团体和各界人士，向所有关心支持张家界建设的国内外朋友、海外侨胞、港澳同胞、台湾同胞，表示衷心的感谢，并致以崇高的敬意！

回顾"三年有改观五年大变样"建设的生动实践，我们不仅取得了丰硕的发展成果，而且积累了弥足珍贵的经验。

第一，办好张家界的事情，必须始终坚持不断解放思想，致力改革开放。观念决定思路，决定一个地方的政治生态、社会生态和发展前途。只有牢牢把握发展这个第一要务，坚持解放思想和改革开放，勇于破除一切阻碍发展的思想观念和体制机制，大胆吸纳世界上一切先进经验和生产力要素，充分运用人类一切优秀的文明成果，不动摇，不徘徊，不折腾，不懈怠，才能卓有成效地推进经济社会大发展。

第二，推进世界旅游精品建设，必须始终坚持从市情出发，不断完善顶层设计。建设世界旅游精品是一项极具挑战性和开创性的综合工程，没有现成的模式和标准。只有准确把握世界现代旅游发展的一般规律，紧密结合后发地区发展特点，适应国内外市场需求，突出张家界旅游特色，不断理清思路、完善顶层设计，才能保证世界旅游精品建设顺利推进、见到实效。

第三，培育壮大产业支撑，必须始终坚持旅游主导，优化结构，转型发展。世界旅游精品建设需要完善的产业体系支撑。只有坚定不移实施旅游带动战略，加快推进旅游转型升级，不断增强和提升旅游发展的关联度和带动力，调整产业结构，转变发展方式，做大做强与之紧密链接的新型工业、文化产业、现代农业和现代服务业，才能不断壮大自我发展的内生动力。

第四，加快发展和加快转型，必须始终坚持项目带动，重点突破。项目既是发展的载体，也是转型的载体。只有把抓发展、促转型的一切工作落实到项目上，抓住一切有利时机、调动一切积极因素，审时度势，踩准节奏，真抓实干，大思维谋划，大手笔动作，高标准建设，才能实现经济社会又好又快发展。

第五，实现富民强市目标，必须始终坚持科学统筹，协调发展。全面协调、统筹兼顾，既是科学发展观的根本要求，也是推进富民强市的内在规律。只有正确处理加快发展与加快转变、经济发展与社会进步、推动发展与改善民生、城市提升与农村发展、经济社会建设与生态环境保护的关系，坚持科学，力求有机统一，才能加快建设全面小康、真正实现富民强市可持续目标。

第六，保障和改善民生是人民政府一切工作的出发点和落脚点，必须始终坚持把发展人民群众的根本利益和解决人民群众的现实问题有机统一起来。我们既要着眼长远，加快完善公共服务体系，让全体人民分享改革发展成果；又要立足当前，把解决好人民群众教育、就业、社会保障、医疗卫生、住房等现实问题摆在突出位置，这样才能使我们事业的成果能及时迅速地惠及全体人民，以极大程度地调动广大人民群众支持我们谋发展、干事业、成大事的积极性。

第七，提高政府公信力和执行力，必须始终坚持依法行政、科学决策和诚信办事。政府的公信力和执行力是我们带领全市各族人民团结奋斗的重要保证。在"五年大变样"的建设进程中，我们围绕建设开放、务实、诚信、效能、廉洁的法治政府和服务型政府，严格执行科学决策制度，大力推进依法行政、阳光行政，聚民心，顺民意，形成了推动经济社会发展的强大合力。

各位代表，过去五年的生动实践，既创造了丰硕的物质成果又积累了宝贵的精神财富，成功地把我市经济社会发展提升到了一个新的层面，在张家界发展的历史进程中写下了辉煌的一页。它对于张家界的发展具有开启性，突破性和鼓舞性。但是，我们面临的困难和问题还不少。在新一轮发展的前启，我们要始终保持清醒的头脑：一是作为后发地区的基本特征还没有根本改变，发展相对不快、不足、不优的问题突出。去年全市人均GDP、人均财政收入、城镇居民人均可支配收入、农民人均纯收入，分别还只相当于全省的64.6%、47.8%、76.7%、65.2%，在全省分别排在第10、8、11、12位。二是经济发展仍处于初级阶段，产业发展面临着做大总量与优化结构的双重压力。比如，休闲度假旅游还没有真正做起来，工业经济才刚刚起步，服务业整体上仍然处于低水平运行；再比如，全市万元GDP财税贡献只有583元，仅为全省的75%；工业化率仅为20.9%，不到全省的一半。三是基础设施的瓶颈制约仍然突出，城市集聚辐射带动功能不强，经济发展缺乏强有力的引擎。城镇化率比全国低了将近10个百分点，中心城区人口和建成区面积分别只有全国地级以上城市平均水平的14.5%和18.4%，全市32个建制镇人口规模大多远远低于国内外公认的适宜发展规模。四是发展不平衡的问题还比较突出，社会事业发展历史欠账较多，城乡差距较大。比如，城乡居民收入比达到3.45倍，比全省高0.51个百分点。按照新的贫困标准，尚有近20万农村人口生活在贫困线以下。五是一些思想观念、工作方法和体制机制不能适应加快发展、加快转型的新任务新要求。一些地方和部门对政策理解不透、跟进不力，出手比别人慢，受制比别人多；一些同志特别是有的领导同志对BT、BOT等新型融资方式不会用、不敢用，甚至怀疑、抵制；一些城区拆迁、施工、治安和政务环境等方面问题还较多，等等。对于这些问题，我们必须高度重视，认真研究并不断加以解决。

二、再干新五年，再创新辉煌，奋力推进我市由旅游产业大市向旅游经济强市迈进

各位代表，"三年有改观五年大变样"目标基本实现，我们已站在了一个崭新的历史起点。展望未来，奋力推进我市由旅游产业大市向旅游经济强市转变，其势已成，其时已至。我们唯有因势利导，奋力拼搏，不断创造新业绩、实现新跨越，才能不辱历史使命，不负全市各族人民的信任和期盼。

纵观当前大势，虽然国际政治经济环境复杂多变，风险明显增多，但世界多极化、经济全球化的趋势没有改变，和平、发展、合作仍然是当今时代潮流。我国经济发展正处于重要战略机遇期和转型加速期，稳增长、调结构与控通胀的压力沉重，发展不平衡、不协调、不可持续的问题突出，但国民经济朝着宏观调控预期方向发展，长期向好的趋势更加稳固，中央将继续实施积极财政政策和稳健货币政策，保持宏观经济政策的连续性和稳定性，增强调控的针对性、灵活性和前瞻性，牢牢把握扩大内需这一战略基点，牢牢把握发展实体经济这一坚实基础，牢牢把握加快改革创新这一强大动力，牢牢把握保障和改善民生这一根本目的，实现经济社会又好又快发展。省第十次党代会作出了全面推进"四化两型"建设，加快实现全面小康的战略部署，把旅游产业、现代服务业纳入战略性新兴产业培育和发展，大力支持武陵山片区区域发展和扶贫攻坚，继续实施大湘西开发战略。我市正处在强基础、调结构、促转型的重要时期，虽然面临诸多挑战，但机遇与挑战同在。国家武陵山片区区域发展与扶贫攻坚规划已经启动实施，必将有效带动我市经济社会发展重大基础设施建设；国家旅游综合改革试点的纵深推进，必将有效促进我市加快转型发展中体制机制和结构性难题的破解；航空口岸扩大开放，必将有效加速我市对外开放和旅游国际化发展进程。特别是"三年有改观五年大变样"的建设实践，为我们谋求更快更好的发展奠定了良好的物质技术基础和思想观念基础。我们面临的挑战，主要是发展内生动力不足、发展要素制约和思想观念滞后。全市上下在市委的统一领导下，应该而且必须以高度的政治责任感、历史使命感和时不我待的紧迫感，进一步明晰工作思路、完善顶层设计，推动全市经济社会发展迈上新的更高更大台阶。

今后一个时期的基本指导思想是：高举中国特色社会主义伟大旗帜，以邓小平理论、"三个代表"重要思想和科学发展观为指导，认真贯彻省第十次党代会全面推进"四化两型"战略部署和市第六次党代会加快推进"五化五建"工作要求，紧扣建设世界旅游精品和富民强市总目标，坚持旅游主导路径，立足"三个发展平台"，抢抓机遇，两轮驱动，率先在武陵山片区区域发展和扶贫攻坚中实现阶段性目标，率先在国家旅游综合改革试点中取得先行先试成果，在全省率先打造"两型社会"，再干新五年，再创新辉煌，奋力推进旅游产业大市向旅游经济强市跨越。

坚持旅游主导路径：就是要坚持"以旅游业为主导，新型工业强市，现代服务业兴市，加快城市建设，发展现代农业，促进扶贫攻坚"的基本思路不动摇。

立足三个发展平台：一是立足武陵山片区区域发展与扶贫攻坚试点平台。这是一个涵盖基础设施、特色产业、社会事业、公共服务、生态环境等各领域的综合性发展平台。《武陵山片区区域发展和扶贫攻坚规划》已将我市两区两县纳入其中，在其"六中心四轴线"经济带格局中，将我市列为中心之一，承担武汉、长株潭经济圈及长江中游城市的辐射和对接，打造国际风景旅游城市；在其"两环四横五纵"交通主通道中，将我市布局在以中心城市为支点的"内环"、以县城为支点的"外环"和"一横""两纵"上，打造区域性综合交通枢纽；在其旅游业"五组团十二精品线路"布局中，将我市定位为整个片区的旅游综合服务中心、对外形象窗口和旅游交通集散中心，有8条精品线路从我市始发。二是立足国家旅游综合改革试点平台。这个平台最大的优势就是"先行先试"。长株潭两型社会试验区，国家给的政策也就是"先行先试"，现在吸引了55家全球500强企业落户。三是立足国际航空口岸平台。这个平台不仅是旅游国际化的助推器，更是临空经济的孵化器。货物报关出口可以促进航空物流、陆路物流、高轻产品制造、商务流通、金融保险等一系列配套产业发展。这三个平台给我们带来了千载难逢的重大发展机遇，向我们展示了广阔的发展空间，我们必须把握好、利用好。

两轮驱动：是加快发展与加快转型二者的有机统一。加快发展，就是要通过改造提升和依托我市已有的产业基础，保持较快的经济增长速度，加快发展步伐，不断缩小同全国全省和同类旅游城市的发展差距；加快转型，就是要力促经济结构调整，实现产业转型升级，促进旅游经营由单一型向复合型转变、经济发展由粗放型向集约型转变、发展方式由以基础设施建设拉动产业发展向以产业发展推进基础设施配套转变，大幅度提高经济发展综合效益。加快发展是加快转型的前提，加快转型是加快发展的内在动力，我们必须抓住规律、科学把握、统筹推进。

三个率先：则是我们"再干新五年再创新辉煌"的重要标杆。树立这三个标杆，虽有较大的竞争压力，但更有良好的现实基础。分析武陵山片区六个中心城市发展实际，目前，我市人均GDP、城乡居民人均收入、城镇化率等指标仅次于黔江区；人均财政收入仅次于黔江区和恩施州。黔江区虽然是正厅级行政单元，但实际涵盖范围只是一个县，与其他5市州的可比性不强。除黔江区外，我市经济发展水平在整个片区处于领先地位。通过新五年的努力，我们完全可以率先在片区发展竞争中脱颖而出。分析国家旅游综合改革试点城市实际，在4个试点城市中，我市是唯一集"世界自然遗产"和"世界地质公园"两大世界顶尖品牌于一身、并拥有两个5A级景区的城市，省委、省政府又专门出台了支持我市改革试点的27条扶持政策，特别是我们在"五年大变样"建设中，致力推进旅游转型升级、创新旅游营销、加强旅游目的地管理，已经积累了不少经验，并得到国家旅游局领导的充分肯定，只要我们进一步解放思想，完善顶层设计，大胆突破，就一定能够在改革试点中率先走出一条成功路子。至于在全省率先打造"两型社会"，这是市第六次党代会已经确立了的奋斗目标。我市属于国家重点生态功能区，目前是全国森林覆盖率最高、单位GDP能耗最低的城市之一，生态优势十分明显，我们完全有条件、更有责任在全省率先打造"两型社会"。

"再干新五年再创新辉煌"的重点任务是：按照市第六次党代会提出的"五化五建"总体要求，着力构建"九大体系"，全面提升我市经济社会发展综合功能。

一是构建以旅游业为主导、结构合理的现代产业体系。在不断提升观光旅游的同时，加快发展休闲度假、商务会展、康体养生等现代旅游业，促进旅游国际化发展。加快文化与旅游的融合，突出文化产业的发展，改革完善"政府主导、市场运作"体制机制，依托旅游优势打造传播湖湘文化的大窗口，依托旅游市场构建对外交流、交融、交锋的大舞台，抢占旅游与文化、传统与时尚融合发展的制高点。充分发挥旅游的带动力和牵引力，扎实推进产业结构调整和产业配套建设，做大做强新型工业、现代服务业和现代农业，形成多元支柱产业支撑。到"十二五"期末，力争全市三次产业结构比调整为10︰33︰57，游客人均消费水平提高35%以上，工业、文化产业增加值占GDP比重分别达到25%和12％，主要经济指标基本达到全省平均水平。

二是构建与科学发展相适应的现代基础设施体系。以打造武陵山区旅游交通枢纽为目标，加快构建高速公路、铁路、航空、水运及城市交通无缝对接的现代立体交通网络，形成与重庆、武汉、贵阳等中心城市"五小时交通经济圈"。以增强安全可靠、清洁高效能源供给保障能力为目标，加快推进城乡电网改造和燃气管网建设，加快天然气、页岩气开发，科学开发利用水能、风能、生物质能等清洁能源与可再生能源，力争新增发电装机30万千瓦。以适应城乡发展和产业结构调整为目标，抢抓机遇，加快水利基础设施和重要水源工程建设，全面提高供水安全保障功能和现代化防洪除涝、抗旱减灾功能。
